# Variants Identified in a GWAS Meta-Analysis for Blood Lipids Are Associated with the Lipid Response to Fenofibrate

**DOI:** 10.1371/journal.pone.0048663

**Published:** 2012-10-31

**Authors:** Stella Aslibekyan, Mark O. Goodarzi, Alexis C. Frazier-Wood, Xiaofei Yan, Marguerite R. Irvin, Eric Kim, Hemant K. Tiwari, Xiuqing Guo, Robert J. Straka, Kent D. Taylor, Michael Y. Tsai, Paul N. Hopkins, Stanley G. Korenman, Ingrid B. Borecki, Yii-Der I. Chen, Jose M. Ordovas, Jerome I. Rotter, Donna K. Arnett

**Affiliations:** 1 Department of Epidemiology, School of Public Health, University of Alabama at Birmingham, Birmingham, Alabama, United States of America; 2 Department of Biostatistics, School of Public Health, University of Alabama at Birmingham, Birmingham, Alabama, United States of America; 3 Department of Medicine, Cedars-Sinai Medical Center, Los Angeles, California, United States of America; 4 Medical Genetics Institute, Cedars-Sinai Medical Center, Los Angeles, California, United States of America; 5 Experimental and Clinical Pharmacology Department, College of Pharmacy, School of Public Health, University of Minnesota, Minneapolis, Minnesota, United States of America; 6 Division of Epidemiology and Community Health, School of Public Health, University of Minnesota, Minneapolis, Minnesota, United States of America; 7 Department of Genetics, Washington University in St. Louis, St. Louis, Missouri, United States of America; 8 School of Medicine, University of Utah, Salt Lake City, Utah, United States of America; 9 Department of Medicine, David Geffen School of Medicine, Los Angeles, California, United States of America; 10 Department of Epidemiology, Atherothrombosis and Imaging, Centro Nacional de Investigaciones Cardiovasculares, Madrid, Spain; 11 Instituto Madrileño de Estudios Avanzados Alimentacion, Madrid, Spain; 12 Jean Mayer United States Department of Agriculture Human Nutrition Research Center on Aging, Tufts University, Boston, Massachusetts, United States of America; The Children's Hospital of Philadelphia, United States of America

## Abstract

A recent large-scale meta-analysis of genome-wide studies has identified 95 loci, 59 of them novel, as statistically significant predictors of blood lipid traits; we tested whether the same loci explain the observed heterogeneity in response to lipid-lowering therapy with fenofibrate. Using data from the Genetics of Lipid Lowering Drugs and Diet Network (GOLDN, n = 861) we fit linear mixed models with the genetic markers as predictors and high-density lipoprotein (HDL) cholesterol, low-density lipoprotein (LDL) cholesterol, total cholesterol, and triglyceride concentrations as outcomes. For all four traits, we analyzed both baseline levels and changes in response to treatment with fenofibrate. For the markers that were significantly associated with fenofibrate response, we fit additional models evaluating potential epistatic interactions. All models were adjusted for age, sex, and study center as fixed effects, and pedigree as a random effect. Statistically significant associations were observed between the rs964184 polymorphism near *APOA1* (P-value≤0.0001) and fenofibrate response for HDL and triglycerides. The association was replicated in the Pharmacogenetics of Hypertriglyceridemia in Hispanics study (HyperTG, n = 267). Suggestive associations with fenofibrate response were observed for markers in or near *PDE3A, MOSC1*, *FLJ36070, CETP*, the *APOE-APOC1-APOC4-APOC2*, and *CILP2*. Finally, we present strong evidence for epistasis (P-value for interaction =  0.0006 in GOLDN, 0.05 in HyperTG) between rs10401969 near *CILP2* and rs4420638 in the *APOE-APOC1-APOC4-APOC2* cluster with total cholesterol response to fenofibrate. In conclusion, we present evidence linking several novel and biologically relevant genetic polymorphisms to lipid lowering drug response, as well as suggesting novel gene-gene interactions in fenofibrate pharmacogenetics.

## Introduction

A recent large-scale meta-analysis of genome-wide studies has identified 95 loci, 59 of them novel, to be significantly associated with fasting blood lipid traits, including high density lipoprotein (HDL) cholesterol, low density lipoprotein (LDL) cholesterol, total cholesterol, and triglycerides. Together these variants explain 10–12% of phenotypic variability [1], [2]. However, it remains unknown whether genetic variation at these loci also mediates the effects of lipid-lowering pharmaceutical agents.

One such agent is fenofibrate, a peroxisome proliferator-activated receptor-α (PPAR-α) agonist that is widely used to treat dyslipidemia. Individual response to fenofibrate therapy is highly heterogeneous, suggesting a role for pharmacogenetic predictors [3], [4]. Although prior studies have implicated single nucleotide polymorphisms (SNPs) in genes such as *CYP7A1, PPARA*, and the *APOA1/C3/A4/A5* cluster in lipid response to fenofibrate, their findings explain only a small percentage of outcome variation [5]–[7]. Similarly to other complex traits, the effect of fenofibrate treatment on lipids is likely to have a non-linear genetic architecture, as evidence suggests that triglyceride response in particular is influenced by epistatic interactions in a subset of key polymorphisms [8]. However, reports on changes in other lipid outcomes remain limited to single gene analyses, prompting a comprehensive approach to discovering novel biologically relevant loci. If successfully validated, newly discovered markers could be used as predictive tools to guide clinical treatment decisions [9].

This study investigated whether the previously validated genetic predictors of lipid traits are associated with baseline blood lipids as well as with the change in triglycerides, HDL-, LDL-, and total cholesterol following 3 weeks of fenofibrate therapy in the participants of the Genetics of Lipid Lowering Drugs and Diet Network (GOLDN). For those genetic markers that produced statistically significant associations with the change in lipids, we conducted replication analyses in a cohort of Mexican-Americans treated with fenofibrate: the Pharmacogenetics of Hypertriglyceridemia in Hispanics study (HyperTG, n = 267). Additionally, for the top fenofibrate-related markers, we evaluated all possible pairwise epistatic combinations and tested the statistically significant interactions in the HyperTG population, providing further insights into the biology of lipid response to fenofibrate.

## Results

Of the 95 loci originally validated as blood lipid concentration predictors, 6 were not associated with the outcomes in Caucasian populations and thus excluded from subsequent analyses, as the GOLDN cohort is entirely comprised of European Americans (Table S1). Additionally, genotype information for the rs9411489 marker in the *ABO* gene was not available in GOLDN, so we identified and used rs495828 as its proxy (r^2^ = 1.00, D' = 1.00 in the CEU subpopulation of 1000 Genomes). The remaining SNPs were either genotyped or imputed with a high degree of accuracy; out of the 80 imputed SNPs, none had an imputation quality score (R-squared) below 0.3 and only 11 had an imputation quality score below 0.8. Additionally, no evidence of deviation from Hardy-Weinberg equilibrium (P<1×10^−6^) was found for any of the 15 genotyped loci. Finally, none of the examined SNPs were rare (minor allele frequency (MAF) <0.01) in the GOLDN study population (Table S1).

Clinical and demographic characteristics of the GOLDN and HyperTG populations are shown in Table 1. Except for the ethnicity distinction, the discovery and replication cohorts had similar demographic and risk factor profiles. In both cohorts, participants were middle-aged (48±16 years in GOLDN, 41±12 in HyperTG) and evenly split between genders. The mean LDL- and total cholesterol concentrations at baseline were below national averages, suggesting a favorable lipoprotein profile in the study population, although the observed serum HDL cholesterol was also slightly below the national average [10]. Following the three-week treatment with fenofibrate, clinically significant changes were observed for all four lipid outcomes (Table 1).

**Table 1:** Clinical and demographic characteristics of the discovery and replication study populations.

Variable (mean/median* ± SD or %)	GOLDN	HyperTG
	N = 861	N = 267
Age, years	48±16	41±12
Sex, % female	50	52
Current smoker, %	8	–
Body mass index, kg/m^2^	28±6	31±4
High density lipoprotein cholesterol, mg/dL		
Baseline	46.65±13.10	41.83±9.39
After fenofibrate treatment	49.47±13.41	46.35±10.51
Low density lipoprotein cholesterol, mg/dL		
Baseline	123.18±31.47	102.25±33.10
After fenofibrate treatment	104.30±31.30	89.10±28.90
Total cholesterol, mg/dL		
Baseline	192.09±39.24	186.17±37.38
After fenofibrate treatment	166.83±34.37	166.27±34.38
Triglycerides, mg/dL		
Baseline	115.00±98.97	198.30±136.69
After fenofibrate treatment	75.50±55.22	122.95±140.68

* Medians were reported for triglycerides only due to skewness in the data.

The top genetic predictors of lipid fenofibrate response are listed in Table 2, with SNPs that were tested but not found to be significant pharmacogenetic predictors summarized in Table S2. The threshold for Bonferroni correction for multiple comparisons was based on 356 tests (86 SNPs x 4 lipid outcomes), which yields an adjusted alpha level of 0.0001. The strongest association in GOLDN, which replicated successfully in HyperTG, was observed between the rs964184 polymorphism near *APOA1* and increased HDL cholesterol and triglycerides following fenofibrate therapy. Additionally, in GOLDN it reached suggestive significance levels for changes in LDL cholesterol, although the magnitude of the corresponding associations was more modest for fenofibrate response than for baseline values. At baseline, rs964184 was associated with triglycerides (Table S3). Additionally, rs3764261 in the *CETP* gene was found to be associated with HDL cholesterol at baseline in GOLDN (Table S3). Overall, the top associated SNPs for each lipoprotein type varied between baseline and response values in either genomic region, magnitude of association (as indicated by a range of F-statistics), or both, suggesting a decreased likelihood of bias due to residual confounding by baseline lipid concentration.

**Table 2:** Genetic polymorphisms associated with lipid response to fenofibrate therapy in GOLDN (n = 861) and HyperTG (n = 267).

SNP (Variant Allele)	Locus	F	Discovery P-value	Replication P-value	Direction of Association Discovery/Replication
**High-density lipoprotein cholesterol**					
rs964184 (G)	*APOA1*	19.8	<0.0001	0.04	Increase/Increase
rs7134375 (A)	*PDE3A*	9.3	0.002	0.75	Increase/NS
**Low-density lipoprotein cholesterol**					
rs3764261 (A)	*CETP*	12.9	0.0004	0.38	Decrease/NS
rs4420638 (G)	*APOE*	10.4	0.001	0.10	Increase/NS
rs964184 (G)	*APOA1*	10.1	0.002	0.42	Increase/NS
rs2642442 (C)	*MOSC1*	4.8	0.03	0.25	Decrease/NS
rs10401969 (C)	*CILP2*	4.1	0.04	0.64	Decrease/NS
**Total cholesterol**					
rs3764261 (A)	*CETP*	7.3	0.007	0.19	Decrease/NS
rs4420638 (G)	*APOE*	6.9	0.009	0.18	Increase/NS
rs492602 (G)	*FLJ36070*	6.1	0.01	0.41	Decrease/NS
rs10401969 (C)	*CILP2*	4.9	0.03	0.99	Decrease/NS
**Triglycerides**					
rs964184 (G)	*APOA1*	15.3	0.0001	0.05	Increase/Increase

Table 3 and Figur S1 summarize the evidence for epistatic interaction for selected genes in GOLDN and HyperTG. Based on a Bonferroni-adjusted alpha level of 0.003 resulting from simultaneously testing 17 pairwise interaction hypotheses, statistically significant epistasis was observed between rs10401969 near *CILP2* and rs4420638 near *APOE* for the total cholesterol outcome. In GOLDN, all cell counts were all greater than 5 participants. However, in the HyperTG cohort, the small sample size resulted in sparse cells; as a result, we have grouped together homozygous mutant and heterozygous genotypes for the replication analysis. In GOLDN, among participants carrying no variant alleles at the *CILP2* locus, the total cholesterol response was slightly attenuated with each copy of the variant *APOE* allele. Among carriers of the minor *CILP2* allele, however, the total cholesterol response was more pronounced, ranging from the ratio of 0.89 for wildtype *APOE* homozygotes to 0.83 for carriers of at least one copy of the variant allele. Although the regression coefficient for the *APOE/CILP2* interaction term also achieved borderline statistical significance in the replication cohort, it is noteworthy that the direction of association between *CILP2* genotypes and the total cholesterol ratio for carriers of at least one copy of the *APOE* variant allele was reversed in HyperTG (Figure S1).

**Table 3:** Suggestive evidence of gene-gene interactions for selected serum lipid concentrations and genotypes.

Joint Genotype	MA1* × MA2	Post-Treatment to Baseline Ratio ± SE (Discovery)	Interaction P-value (Discovery)	Interaction P-value (Replication)
**Total cholesterol**				
rs10401969 and rs4420638	0	0.86±0.007	0.0006**	0.05
	1	0.93±0.04		
	2	0.99±0.04		
	4	1.01±0.10		
**Low density lipoprotein cholesterol**				
rs964184 and rs3764261	0	0.79±0.03	0.004	0.61
	1	0.81±0.02		
	2	0.90±0.02		
	4	1.00±0.06		
rs964184 and rs4420638	0	0.69±0.08	0.02	0.77
	1	0.71±0.06		
	2	0.81±0.02		
	4	0.93±0.13		
rs10401969 and rs4420638	0	0.84±0.01	0.04	NA
	1	0.91±0.07		
	2	0.98±0.07		
	4	0.89±0.18		
rs964184 and rs2642442	0	0.81±0.02	0.07	0.14
	1	0.86±0.02		
	2	0.89±0.02		
	4	0.96±0.06		

* Minor allele count.

** Survives Bonferroni correction for 17 hypotheses.

Similar interactions were observed between rs10401969 and rs4420638 for the LDL cholesterol response outcome, consistent with the high correlation between the two phenotypes. Additionally, there was evidence of suggestive interactions between rs3764261 in *CETP* gene rs964184 near *APOA1* for LDL cholesterol. Pairwise interactions between other SNPs listed in Table 3 were tested but did not approach statistical significance. Both main effects and gene-gene interaction results were robust to sensitivity analyses, in which the fenofibrate response outcome was modeled as the difference between post-treatment and baseline lipid concentrations, adjusted for the baseline value (data not shown).

## Discussion

In this study, we evaluated whether genetic loci with replicated associations with circulating blood lipids could also be used as markers of lipid response to fenofibrate therapy. In addition to replicating several of the reported gene-phenotype associations in our study population at baseline, we also present evidence suggesting that several polymorphisms, most notably in the *CETP* and the *APOA1* genes, are associated with treatment-related changes in lipoproteins and triglycerides. Finally, we present data in support of interactions between variants in *APOE* and *CILP2* in total cholesterol models concentrations.

The rs964184 locus near the *APOA1* gene emerged as the most consistent predictor of lipid fenofibrate response, showing statistically significant associations for changes in HDL cholesterol and triglycerides, and approaching statistical significance for LDL cholesterol. The HDL result replicates findings from a recent analysis of genetic predictors of response to fenofibrate and statins among individuals with atherogenic dyslipidemia, which implicated the rs964184 minor allele in lipid profile improvements following combination therapy [11]. However, the association between rs964184 and triglycerides was only observed before treatment, while in GOLDN data it was associated with both baseline concentration and fenofibrate response. This discrepancy could be explained by differences in baseline characteristics between study populations, as evidenced by better metabolic health of the GOLDN participants, and/or differences in treatment regimen. The baseline association with triglycerides seen in GOLDN was also replicated among dyslipidemic individuals of Mexican descent, a population in which the evidence for association is especially strong (odds ratio for hypertriglyceridemia = 1.74) and the MAF at the rs964184 locus exceeds that in Caucasians by more than twofold, thus accounting for a large degree of outcome variability [12].

In the response models, associations approaching statistical significance were also observed for rs7134375 in the *PDE3A* gene and HDL cholesterol; rs2642442 in *MOSC1* for LDL cholesterol; rs492602 in *FLJ36070* for total cholesterol; and rs3764261 in *CETP*, rs4420638 near the *APOE-APOC1-APOC4-APOC2* cluster, and rs10401969 near *CILP2* for both LDL- and total cholesterol. The observed association with the *CETP* variant, is concordant with the extensive evidence demonstrating that fenofibrate improves lipid profile by reducing cholesteryl ester transfer, a process dependent on the product of *CETP*, from HDL to very low density lipoprotein (VLDL) in both mice [13] and humans [14]. Although a previous study from our group investigated *APOE* ε2/ε3/ε4 genotypes as a potential predictor of triglyceride response to fenofibrate and found no associations [15], the rs492602 polymorphism examined in our analysis represents a distinct genetic variant within the *APOE-APOC1-APOC4-APOC2* cluster and a novel locus for fenofibrate response. Similarly, SNPs in *PDE3A, MOSC1, CILP2*, and *FLJ36070* have not been previously linked to pharmacological changes in lipids and warrant further investigations in independent populations.

The strongest evidence for epistasis was observed between rs10401969 near *CILP2* and rs4420638 near the *APOE-APOC1-APOC4-APOC2* cluster for both LDL- and total cholesterol. The two unlinked variants are located on the opposing arms of chromosome 19. To the best of our knowledge, our study is the first to evaluate their interaction, as the relevance of *CILP2* to lipid metabolism has only recently been established [16]. However, the observed statistical interaction is biologically plausible, as *APOE* variants have previously been shown to interact with other lipid-related genes to influence therapeutic response [17]. Suggestive epistasis was also observed between rs3764261 in *CETP* and the rs964184 locus near *APOA1* in the LDL cholesterol models, which did not reach statistical significance but is likely to have functional relevance. Specifically, prior experiments conducted in mice showed that *CETP* expression reversed the HDL cholesterol lowering action of a pregnane X receptor agonist, which was shown to occur through decreased expression of the *APOA1* homolog [18]. Future replication studies, as well as experiments using CETP knockout animals treated with fenofibrate, could support the validity of our findings and further elucidate the epistatic mechanisms underlying response to lipid-lowering drugs.

The results of our study should be interpreted in the context of several important considerations. First, only a subset of our findings were evaluated and replicated in an independent population. Reasons for such partial non-replication are manifold and could include effect modification by ancestry or other baseline factors, lack of statistical power in the smaller replication cohort, variation in fenofibrate regimen (3 weeks vs. 8 weeks) and compliance, and others. In light of these concerns, we have taken great care to reduce the likelihood of false positive findings, namely by using extremely conservative multiple testing adjustments and presenting evidence of biological relevance of the examined loci. Additionally, stringent genotyping quality control procedures implemented in GOLDN, high imputation quality scores (Table S1), and the absence of implausibly low P-values all serve to indirectly corroborate the validity of our findings [19].

Second, we chose a candidate gene approach rather than conduct a genome-wide association study to enable biologically informed analyses of gene-gene interactions and single marker associations. Given that effect of any given genetic marker on a complex trait like fenofibrate response is likely to be small, the sample size of both discovery and replication cohorts would have been insufficient to conduct a genome-wide search for interactions, or possibly even to reveal all relevant single markers. Therefore, we have built our research question upon *a priori* understanding of fenofibrate pharmacogenetics as well as robust evidence of genetic determinants of lipid levels published by Teslovich et al. [1]. For similar reasons, we did not evaluate any higher-order interactions, which could potentially contribute to the genetic architecture of the outcome and either mask or alter the effects of the examined genetic variants [20].

Third, the loci identified in the original meta-analysis represent only common variants, even though recent evidence highlights the importance of rare variants in the genetic architecture of lipid traits [21]. Fourth, the baseline lipid profile of our discovery population is more favorable than in those with true indication for fenofibrate, limiting the generalizability of our findings. Finally, the current study can offer only limited mechanistic insights into the relationship between rs964184 near *APOA1* and the lipid response to fenofibrate. Future studies may consider investigating the association between variation at that locus and fenofibrate-induced changes in plasma apolipoproteins (specifically apoA-I, apoC-III, apoA-IV, or apoA-V) to elucidate the underlying biological pathways.

In conclusion, we have successfully confirmed associations between key loci and circulating lipids in baseline as well as identified novel genetic predictors of lipid response to fenofibrate. We also found biologically relevant interactions between polymorphisms near *CILP2* and the *APOE-APOC1-APOC4-APOC2* cluster in the LDL- and total cholesterol models, adding to the body of evidence suggesting that genetic factors have an important and nuanced role in determining effectiveness of lipid-lowering therapy.

## Materials and Methods

### Ethics Statement

Participants provided written informed consent and all research was conducted according to the principles outlined in the Declaration of Helsinki. The protocol was approved by the Institutional Review Boards at the University of Minnesota, University of Utah, Tufts University/New England Medical Center, UCLA, Cedars-Sinai Medical Center, and the University of Alabama at Birmingham.

### Discovery Study Population

Detailed descriptions of methods, including genotyping and circulating lipids measurement, are presented in Text S1. Briefly, the National Heart, Lung, and Blood Institute GOLDN study, described in detail in previous publications [13], [22], was designed to identify genetic determinants of lipid response to daily treatment with 160 mg of micronized fenofibrate over the course of three weeks. GOLDN recruited European American pedigrees with at least two siblings from the genetically homogeneous communities of Minneapolis, MN, and Salt Lake City, UT. For the fenofibrate portion of the trial, participants discontinued the use of lipid-lowering agents for at least 4 weeks, fasted for at least 8 hours prior to study visits, and abstained from alcohol for at least 24 hours before each study visit. Circulating blood lipids were measured at baseline and following the three-week treatment period. A total of 906,600 SNPs were genotyped using the Affymetrix Genome-Wide Human 6.0 array and the Birdseed calling algorithm [23]. After quality control exclusions, 584,029 typed SNPs remained. We subsequently imputed untyped SNPs using Human Genome Build 36 as referent, and created a hybrid dataset that included a total of 2,543,887 SNPs (584,029 typed and 1,959,858 imputed) [23]. Population stratification was assessed using principal component analysis and found to be limited in our study population [23].

Participants were excluded from the analysis if they if they were missing outcome data, yielding n = 861 for the single marker analyses. Of those, participants that were missing genotype information at specific loci were excluded from the respective analyses, which explains the reduced sample size in the epistasis models. Outcomes were defined as: 1) baseline treatment plasma concentrations of HDL-, LDL-, total cholesterol and triglycerides; 2) post-treatment/baseline ratios of plasma concentrations for the four outcomes as defined above. Log- transformations were carried out for triglyceride ratios as the variable was not normally distributed. Sensitivity analyses were conducted defining the outcome as the difference in post- and pre-fenofibrate treatment lipid concentrations with the model adjusted for baseline concentrations, as well as with using non-HDL cholesterol as an outcome.

Linear mixed models were fit to evaluate the associations between single markers previously linked to lipid levels in European individuals [1] and the outcomes, adjusted for sex, age, and center as fixed effects, and pedigree as a random effect. The additive assumption was used to model genotypes. The SNPs that showed a suggestive or statistically significant association with the outcomes (nominal P-value <0.05) were included in subsequent interaction analyses, in which a multiplicative term between any two given SNPs was included into the regression model as described above. For the single marker analyses, P-values were adjusted for multiple testing using the Bonferroni approach based on 356 hypotheses (86 SNPs x 4 lipid outcomes), with the final adjusted alpha level of 0.0001. For the interaction analyses, the Bonferroni correction was based on the 17 pairwise tests, yielding a statistical significance threshold of 0.003. The chosen corrections are likely to be conservative given the highly correlated nature of the lipid phenotypes. All association analyses were carried out in SAS v. 9.2 (Cary, NC).

### Replication Study Population

HyperTG recruited 350 Hispanic-American participants from Los Angeles, CA for a pharmacogenetic study of the response to fenofibrate (160 mg per day for 8 weeks). Of the 350 subjects, 267 had complete total cholesterol, HDL cholesterol, and triglyceride measurements and genotype data and were analyzed in this study for the replication of the 7 SNP associations. Pedigree structure was examined and potential population stratification was assessed using principal component analysis.

Outcomes were defined as: 1) baseline treatment plasma concentrations of HDL-, LDL-, total cholesterol and triglycerides; 2) post-treatment/baseline ratios of plasma concentrations for the four outcomes as defined above. Participants were excluded from the LDL cholesterol analysis if their triglyceride value was larger than 400 mg/dL. Log- transformations were carried out for baseline triglyceride levels and the four treatment/baseline ratios because the raw data were not normally distributed. Sensitivity analyses were conducted defining the outcome as the difference in post- and pre-fenofibrate treatment lipid concentrations with the model adjusting for and baseline concentrations, as well as with using non-HDL cholesterol as an outcome.

Linear mixed models were fit to evaluate the associations between each of the 7 single SNPs identified in GOLDN and the outcomes, adjusted for sex, age, and pedigree as a random effect. The additive assumption was used to model genotypes. In the replication of the interaction analyses of the four pairs of markers rs964184*rs3764261, rs964184*rs4420638, rs964184*rs2642442 and rs10401969*rs4420638, a multiplicative term between the two SNPs was included into the regression model. All association analyses were carried out in SAS v. 9.2 (Cary, NC).

## Supporting Information

Figure S1 **Evidence of epistatic interaction between rs10401969 and rs4420638 in total cholesterol response to fenofibrate in (A) the discovery GOLDN cohort (n = 861) and (B) replication HyperTG cohort (n = 267).**
(DOCX)

Table S1 **Characteristics of loci identified by Teslovich et al. to be associated with plasma lipids in the GOLDN population.**
(DOC)

Table S2 **Genetic polymorphisms identified by Teslovich et al. that were not found to be associated with lipid response to fenofibrate therapy in GOLDN (n = 861).**
(DOC)

Table S3 **Associations between selected genetic polymorphisms and lipid concentrations at baseline.**
(DOC)

Text S1 **Detailed description of genotyping and circulating blood lipid measurement in the GOLDN and HyperTG studies.**
(DOC)
