# Supplementary figures and images for: Variants Identified in a GWAS Meta-Analysis for Blood Lipids Are Associated with the Lipid Response to Fenofibrate
Source: PLoS One. 2012 Oct 31;7(10):e48663. doi: 10.1371/journal.pone.0048663 (PMC3485381; doi:10.1371/journal.pone.0048663)

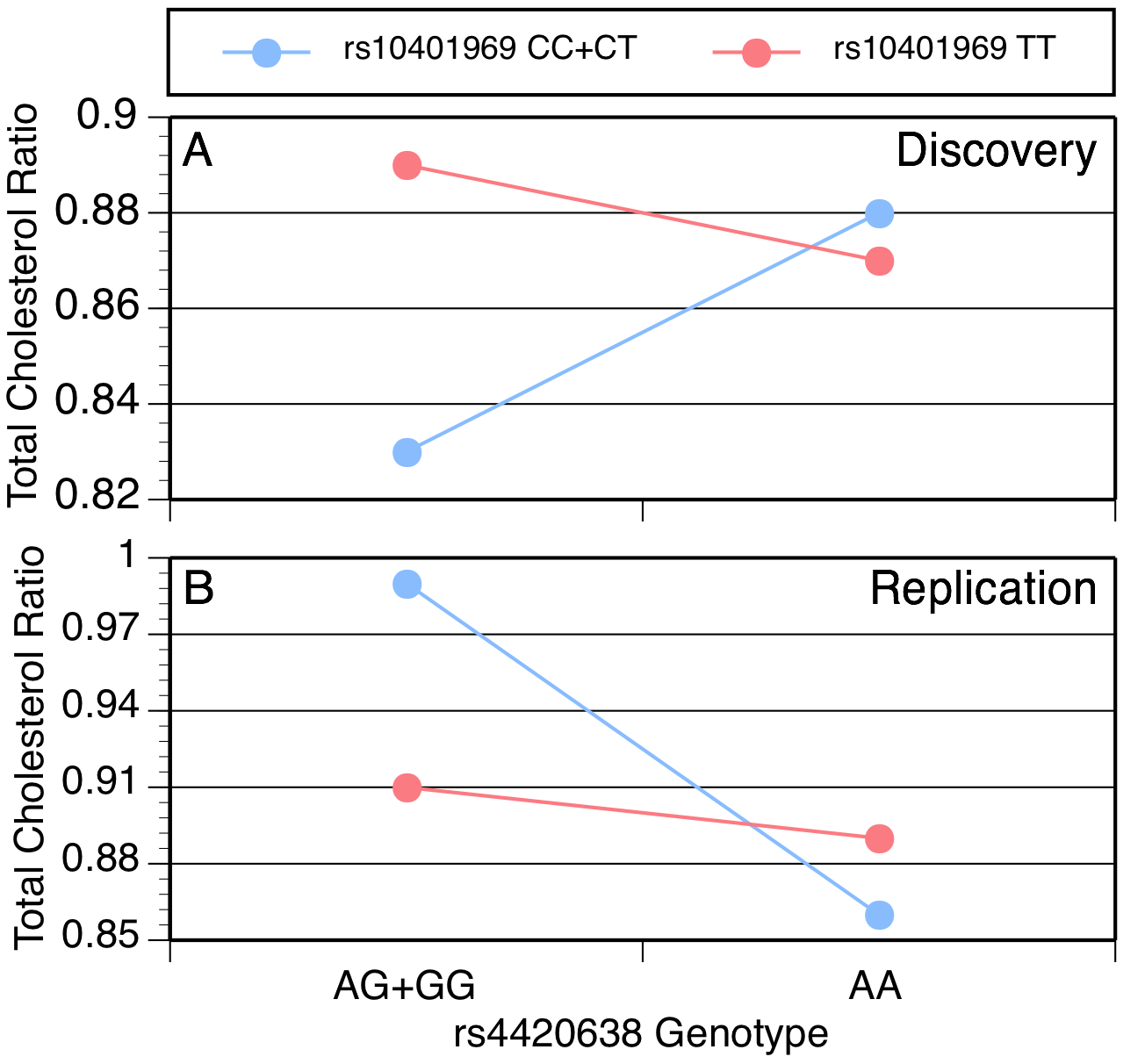

Supplement: Figure S1 — Evidence of epistatic interaction between rs10401969 and rs4420638 in total cholesterol response to fenofibrate in (A) the discovery GOLDN cohort (n = 861) and (B) replication HyperTG cohort (n = 267). (DOCX) [file pone.0048663.s001.docx]
