# Supplementary material for: Variants Identified in a GWAS Meta-Analysis for Blood Lipids Are Associated with the Lipid Response to Fenofibrate
Source: PLoS One. 2012 Oct 31;7(10):e48663. doi: 10.1371/journal.pone.0048663 (PMC3485381; doi:10.1371/journal.pone.0048663)
Supplement: Table S1 — Characteristics of loci identified by Teslovich et al. to be associated with plasma lipids in the GOLDN population. (DOC) [file pone.0048663.s002.doc]

| **SNP** | **Locus** | **Chr** | **Alleles** | **MAF** | **Imputed?** | **r^2^** | **P(HWE)** |
| --- | --- | --- | --- | --- | --- | --- | --- |
| rs12027135 | *LDLRAP1* | 1 | T/A | 0.47 | Y | 0.99 | -- |
| rs4660293 | *PABPC4* | 1 | A/G | 0.25 | N | -- | 1.00 |
| rs2479409 | *PCSK9* | 1 | A/G | 0.35 | N | -- | 0.14 |
| rs2131925 | *ANGPTL3* | 1 | T/G | 0.36 | Y | 1.00 | -- |
| rs7515577 | *EVIS* | 1 | A/C | 0.20 | N | -- | 0.76 |
| rs629301 | *SORT1* | 1 | T/G | 0.20 | N | -- | 0.05 |
| rs1689800 | *ZNF648* | 1 | A/G | 0.33 | Y | 0.97 | -- |
| rs2642442 | *MOSC1* | 1 | T/C | 0.30 | N | -- | 0.67 |
| rs4846914 | *GALNT2* | 1 | A/G | 0.41 | N | -- | 0.46 |
| rs514230 | *IRF2BP2* | 1 | T/A | 0.47 | Y | 0.99 | -- |
| rs1367117 | *APOB* | 2 | G/A | 0.33 | Y | 0.95 | -- |
| rs1042034 | *APOB* | 2 | T/C | 0.22 | Y | 1.00 | -- |
| rs1260326 | *GCKR* | 2 | C/T | 0.41 | Y | 0.96 | -- |
| rs4299376 | *ABCG5/8* | 2 | T/G | 0.32 | Y | 0.99 | -- |
| rs7570971 | *RAB3GAP1* | 2 | C/A | 0.35 | Y | 0.97 | -- |
| rs10195252 | *COBLL1* | 2 | T/C | 0.39 | Y | 0.97 | -- |
| rs12328675 | *COBLL1* | 2 | T/C | 0.12 | Y | 0.97 | -- |
| rs2972146 | *IRS1* | 2 | T/G | 0.35 | Y | 1.00 | -- |
| rs2290159 | *RAF1* | 3 | G/C | 0.24 | Y | 1.00 | -- |
| rs645040 | *MSL2L1* | 3 | T/G | 0.19 | N | -- | 0.39 |
| rs442177 | *KLHL8* | 4 | T/G | 0.42 | Y | 0.99 | -- |
| rs13107325 | *SLC39A8* | 4 | C/T | 0.09 | Y | 0.60 | -- |
| *rs6450176 | *ARL15* | 5 | G/A | 0.22 | Y | 0.95 | -- |
| rs9686661 | *MAP3K1* | 5 | C/T | 0.19 | Y | 0.96 | -- |
| rs12916 | *HMGCR* | 5 | T/C | 0.38 | Y | 0.97 | -- |
| rs6882076 | *TIMD4* | 5 | C/T | 0.35 | Y | 1.00 | -- |
| rs3757354 | *MYLIP* | 6 | C/T | 0.23 | Y | 0.83 | -- |
| rs1800562 | *HFE* | 6 | G/A | 0.07 | N | -- | 1.00 |
| rs3177928 | *HLA* | 6 | G/A | 0.12 | N | -- | 0.52 |
| rs2247056 | *HLA* | 6 | C/T | 0.26 | Y | 1.00 | -- |
| rs2814944 | *C6orf106* | 6 | G/A | 0.14 | N | -- | 0.22 |
| *rs2814982 | *C6orf106* | 6 | C/T | 0.09 | Y | 0.72 | -- |
| rs9488822 | *FRK* | 6 | A/T | 0.39 | Y | 0.92 | -- |
| rs605066 | *CITED2* | 6 | T/C | 0.44 | Y | 1.00 | -- |
| rs1564348 | *LPA* | 6 | T/C | 0.16 | N | -- | 0.52 |
| rs1084651 | *LPA* | 6 | G/A | 0.17 | Y | 0.95 | -- |
| rs12670798 | *DNAH11* | 7 | T/C | 0.23 | Y | 0.99 | -- |
| rs2072183 | *NPC1L1* | 7 | G/C | 0.27 | Y | 0.46 | -- |
| rs13238203 | *TYW1B* | 7 | C/T | 0.05 | Y | 0.76 | -- |
| rs17145738 | *MLXIPL* | 7 | C/T | 0.13 | N | -- | 0.37 |
| rs4731702 | *KLF14* | 7 | C/T | 0.50 | Y | 1.00 | -- |
| rs9987289 | *PPP1R3B* | 8 | G/A | 0.11 | Y | 0.98 | -- |
| *rs11776767 | *PINX1* | 8 | G/C | 0.35 | Y | 0.99 | -- |
| *rs1495741 | *NAT2* | 8 | A/G | 0.27 | Y | 1.00 | -- |
| rs12678919 | *LPL* | 8 | A/G | 0.11 | Y | 0.92 | -- |
| rs2081687 | *CYP7A1* | 8 | C/T | 0.34 | Y | 0.99 | -- |
| rs2293889 | *TRPS1* | 8 | G/T | 0.43 | Y | 1.00 | -- |
| rs2737229 | *TRPS1* | 8 | A/C | 0.30 | Y | 0.97 | -- |
| rs2954029 | *TRIB1* | 8 | A/T | 0.48 | Y | 1.00 | -- |
| rs11136341 | *PLEC1* | 8 | A/G | 0.38 | Y | 0.70 | -- |
| rs581080 | *TTC39B* | 9 | C/G | 0.17 | Y | 0.93 | -- |
| rs1883025 | *ABCA1* | 9 | C/T | 0.25 | Y | 0.97 | -- |
| **rs495828 | *ABO* | 9 | G/T | 0.26 | Y | 0.99 | -- |
| rs10761731 | *JMJD1C* | 10 | A/T | 0.41 | Y | 1.00 | -- |
| rs2068888 | *CYP26A1* | 10 | G/A | 0.46 | Y | 0.80 | -- |
| rs2255141 | *GPAM* | 10 | G/A | 0.28 | Y | 1.00 | -- |
| rs2923084 | *AMPD3* | 11 | A/G | 0.19 | N | -- | 0.08 |
| rs10128711 | *SPTY2D1* | 11 | C/T | 0.70 | N | -- | 0.41 |
| rs3136441 | *LRP4* | 11 | T/C | 0.13 | Y | 0.99 | -- |
| rs174546 | *FADS1-2-3* | 11 | C/T | 0.36 | Y | 1.00 | -- |
| rs964184 | *APOA1* | 11 | C/G | 0.13 | N | -- | 0.38 |
| rs7941030 | *UBASH3B* | 11 | T/C | 0.43 | Y | 0.97 | -- |
| rs11220462 | *ST3GAL4* | 11 | G/A | 0.15 | Y | 0.94 | -- |
| rs7134375 | *PDE3A* | 11 | C/A | 0.40 | Y | 0.90 | -- |
| rs11613352 | *LRP1* | 12 | C/T | 0.26 | Y | 1.00 | -- |
| rs7134594 | *MVK* | 12 | T/C | 0.49 | Y | 1.00 | -- |
| rs11065987 | *BRAP* | 12 | A/G | 0.41 | Y | 0.95 | -- |
| rs1169288 | *HNF1A* | 12 | A/C | 0.31 | Y | 0.97 | -- |
| rs4759375 | *SBNO1* | 12 | C/T | 0.07 | Y | 0.88 | -- |
| *rs4765127 | *ZNF664* | 12 | G/T | 0.33 | N | -- | 0.24 |
| rs838880 | *SCARB1* | 12 | T/C | 0.35 | N | -- | 0.42 |
| rs8017377 | *NYNRIN* | 14 | G/A | 0.47 | Y | 0.64 | -- |
| rs2412710 | *CAPN3* | 15 | G/A | 0.02 | Y | 0.97 | -- |
| rs2929282 | *FRMD5* | 15 | A/T | 0.04 | T | 0.98 | -- |
| rs1532085 | *LIPC* | 15 | G/A | 0.37 | Y | 0.98 | -- |
| rs2652834 | *LACTB* | 15 | G/A | 0.20 | Y | 0.91 | -- |
| rs11649653 | *CTF1* | 16 | C/G | 0.38 | N | -- | 0.26 |
| rs3764261 | *CETP* | 16 | C/A | 0.34 | Y | 0.61 | -- |
| rs16942887 | *LCAT* | 16 | G/A | 0.12 | Y | 1.00 | -- |
| rs2000999 | *HPR* | 16 | G/A | 0.22 | Y | 0.98 | -- |
| rs2925979 | *CMIP* | 16 | C/T | 0.31 | Y | 0.98 | -- |
| rs11869286 | *STARD3* | 17 | C/G | 0.31 | N | -- | 0.57 |
| rs7206971 | *OSBPL7* | 17 | G/A | 0.48 | Y | 0.99 | -- |
| rs4148008 | *ABCA8* | 17 | C/G | 0.32 | Y | 0.99 | -- |
| rs4129767 | *PGS1* | 17 | A/G | 0.50 | Y | 0.97 | -- |
| rs7241918 | *LIPG* | 18 | T/G | 0.22 | Y | 1.00 | -- |
| rs12967135 | *MC4R* | 18 | G/A | 0.25 | Y | 1.00 | -- |
| rs7255436 | *ANGPTL4* | 19 | A/C | 0.44 | Y | 0.54 | -- |
| rs6511720 | *LDLR* | 19 | G/T | 0.12 | Y | 0.59 | -- |
| rs737337 | *LOC55908* | 19 | T/C | 0.09 | Y | 0.91 | -- |
| rs10401969 | *CILP2* | 19 | T/C | 0.07 | N | -- | 0.60 |
| rs4420638 | *APOE* | 19 | A/G | 0.21 | N | -- | 0.57 |
| rs439401 | *APOE* | 19 | C/T | 0.27 | Y | 0.31 | -- |
| rs492602 | *FLJ36070* | 19 | A/G | 0.46 | Y | 0.78 | -- |
| rs386000 | *LILRA3* | 19 | G/C | 0.21 | Y | 0.91 | -- |
| rs2277862 | *ERGIC3* | 20 | C/T | 0.13 | Y | 0.99 | -- |
| *rs2902940 | *MAFB* | 20 | A/G | 0.30 | Y | 0.99 | -- |
| rs6029526 | *TOP1* | 20 | T/A | 0.44 | Y | 0.99 | -- |
| rs1800961 | *HNF4A* | 20 | C/T | 0.04 | N | -- | 1.00 |
| rs6065906 | *PLTP* | 20 | T/C | 0.15 | Y | 0.96 | -- |
| rs181362 | *UBE2L3* | 22 | C/T | 0.12 | Y | 1.00 | -- |
| rs5756931 | *PLA2G6* | 22 | T/C | 0.38 | Y | 0.94 | -- |

***Not found to be associated with lipids in Caucasians by Teslovich et al.

**One of the markers (rs9411489 in *ABO*) was not available in GOLDN, so we used rs495828 as a proxy (r^2^= 1.00, D’=1.00 in CEU population of 1000 Genomes)
