# Supplementary material for: Variants Identified in a GWAS Meta-Analysis for Blood Lipids Are Associated with the Lipid Response to Fenofibrate
Source: PLoS One. 2012 Oct 31;7(10):e48663. doi: 10.1371/journal.pone.0048663 (PMC3485381; doi:10.1371/journal.pone.0048663)
Supplement: Table S2 — Genetic polymorphisms identified by Teslovich et al. that were not found to be associated with lipid response to fenofibrate therapy in GOLDN (n = 861). (DOC) [file pone.0048663.s003.doc]

| **SNP** | **Locus** | **Alleles** | **F** | | **P-value** | |
| --- | --- | --- | --- | --- | --- | --- |
| **HDL Cholesterol** | | | | | | |
| rs1042034 | *APOB* | T/C | | 0.12 | | 0.73 |
| rs1084651 | *LPA* | G/A | | 1.52 | | 0.22 |
| rs11613352 | *LRP1* | C/T | | 0.84 | | 0.36 |
| rs11869286 | *STARD3* | C/G | | 0.44 | | 0.51 |
| rs12328675 | *COBLL1* | T/C | | <0.01 | | 0.96 |
| rs12678919 | *LPL* | A/G | | 0.60 | | 0.44 |
| rs12967135 | *MC4R* | G/A | | 0.17 | | 0.68 |
| rs13107325 | *SLC39A8* | C/T | | 3.79 | | 0.05 |
| rs1532085 | *LIPC* | G/A | | 0.15 | | 0.69 |
| rs1689800 | *ZNF648* | A/G | | 0.02 | | 0.90 |
| rs16942887 | *LCAT* | G/A | | 0.18 | | 0.67 |
| rs17145738 | *MLXIPL* | C/T | | 0.65 | | 0.42 |
| rs174546 | *FADS1-2-3* | C/T | | 2.25 | | 0.13 |
| rs1800961 | *HNF4A* | C/T | | 0.08 | | 0.78 |
| rs181362 | *UBE2L3* | C/T | | 0.30 | | 0.58 |
| rs1883025 | *ABCA1* | C/T | | 0.46 | | 0.50 |
| rs2293889 | *TRPS1* | G/T | | 0.01 | | 0.90 |
| rs2652834 | *LACTB* | G/A | | 2.99 | | 0.08 |
| rs2814944 | *C6orf106* | G/A | | 0.36 | | 0.55 |
| rs2923084 | *AMPD3* | A/G | | 0.27 | | 0.61 |
| rs2925979 | *CMIP* | C/T | | 0.16 | | 0.69 |
| rs2954029 | *TRIB1* | A/T | | 0.05 | | 0.82 |
| rs2972146 | *IRS1* | T/G | | 0.14 | | 0.71 |
| rs3136441 | *LRP4* | T/C | | <0.01 | | 0.95 |
| rs3764261 | *CETP* | C/A | | <0.01 | | 0.96 |
| rs386000 | *LILRA3* | G/C | | 0.38 | | 0.54 |
| rs4129767 | *PGS1* | A/G | | 1.88 | | 0.17 |
| rs4148008 | *ABCA8* | C/G | | 2.79 | | 0.10 |
| rs4420638 | *APOE* | A/G | | 0.06 | | 0.81 |
| rs4660293 | *PABPC4* | A/G | | 0.31 | | 0.58 |
| rs4731702 | *KLF14* | C/T | | 1.08 | | 0.30 |
| rs4759375 | *SBNO1* | C/T | | 0.03 | | 0.85 |
| rs4846914 | *GALNT2* | A/G | | 3.71 | | 0.05 |
| rs581080 | *TTC39B* | C/G | | <0.01 | | 0.96 |
| rs605066 | *CITED2* | T/C | | 1.75 | | 0.19 |
| rs6065906 | *PLTP* | T/C | | 0.25 | | 0.62 |
| rs7134594 | *MVK* | T/C | | 0.38 | | 0.54 |
| rs7241918 | *LIPG* | T/G | | 3.60 | | 0.06 |
| rs7255436 | *ANGPTL4* | A/C | | 0.28 | | 0.60 |
| rs737337 | *LOC55908* | T/C | | 1.38 | | 0.24 |
| rs838880 | *SCARB1* | T/C | | 0.05 | | 0.82 |
| rs9987289 | *PPP1R3B* | G/A | | 0.54 | | 0.46 |
| **LDL Cholesterol** | | | | | | |
| rs11065987 | *BRAP* | A/G | | 0.89 | | 0.35 |
| rs11136341 | *PLEC1* | A/G | | 1.8 | | 0.18 |
| rs11220462 | *ST3GAL4* | G/A | | 0.15 | | 0.70 |
| rs1169288 | *HNF1A* | A/C | | 0.49 | | 0.48 |
| rs12027135 | *LDLRAP1* | T/A | | 1.68 | | 0.20 |
| rs12670798 | *DNAH11* | T/C | | 0.17 | | 0.68 |
| rs12916 | *HMGCR* | T/C | | 3.8 | | 0.05 |
| rs1367117 | *APOB* | G/A | | 0.08 | | 0.78 |
| rs1564348 | *LPA* | T/C | | 0.08 | | 0.77 |
| rs174546 | *FADS1-2-3* | C/T | | 0.81 | | 0.37 |
| rs1800562 | *HFE* | G/A | | 1.8 | | 0.18 |
| rs2000999 | *HPR* | G/A | | 0.4 | | 0.53 |
| rs2072183 | *NPC1L1* | G/C | | 2.76 | | 0.10 |
| rs2081687 | *CYP7A1* | C/T | | 0.16 | | 0.69 |
| rs2131925 | *ANGPTL3* | T/G | | 1.82 | | 0.18 |
| rs2255141 | *GPAM* | G/A | | 0.06 | | 0.81 |
| rs2479409 | *PCSK9* | A/G | | 0.01 | | 0.91 |
| rs3177928 | *HLA* | G/A | | 2.16 | | 0.14 |
| rs3757354 | *MYLIP* | C/T | | 0.25 | | 0.62 |
| rs4299376 | *ABCG5/8* | T/G | | 0.02 | | 0.90 |
| rs495828 | *ABO* | G/T | | 0.14 | | 0.71 |
| rs514230 | *IRF2BP2* | T/A | | 0.15 | | 0.70 |
| rs6029526 | *TOP1* | T/A | | 0.81 | | 0.37 |
| rs629301 | *SORT1* | T/G | | 1.47 | | 0.23 |
| rs645040 | *MSL2L1* | T/G | | 0.63 | | 0.43 |
| rs6511720 | *LDLR* | G/T | | <0.01 | | 0.95 |
| rs6882076 | *TIMD4* | C/T | | 0.25 | | 0.61 |
| rs7206971 | *OSBPL7* | G/A | | 1.42 | | 0.23 |
| rs7941030 | *UBASH3B* | T/C | | 1.79 | | 0.18 |
| rs8017377 | *NYNRIN* | G/A | | 0.01 | | 0.94 |
| rs9488822 | *FRK* | A/T | | 0.66 | | 0.42 |
| rs9987289 | *PPP1R3B* | G/A | | 2.66 | | 0.10 |
| **Total Cholesterol** | | | | | | |
| rs10128711 | *SPTY2D1* | C/T | | 0.25 | | 0.61 |
| rs11065987 | *BRAP* | A/G | | 0.14 | | 0.71 |
| rs11136341 | *PLEC1* | A/G | | 0.14 | | 0.71 |
| rs11220462 | *ST3GAL4* | G/A | | 0.26 | | 0.61 |
| rs12027135 | *LDLRAP1* | T/A | | 0.3 | | 0.58 |
| rs1260326 | *GCKR* | C/T | | 3.02 | | 0.08 |
| rs12670798 | *DNAH11* | T/C | | 0.85 | | 0.36 |
| rs12916 | *HMGCR* | T/C | | 0.47 | | 0.50 |
| rs1367117 | *APOB* | G/A | | 0.62 | | 0.43 |
| rs1532085 | *LIPC* | G/A | | <0.01 | | 0.94 |
| rs1564348 | *LPA* | T/C | | 0.72 | | 0.40 |
| rs174546 | *FADS1-2-3* | C/T | | 0.36 | | 0.55 |
| rs1800562 | *HFE* | G/A | | 2.17 | | 0.14 |
| rs1800961 | *HNF4A* | C/T | | 0.92 | | 0.34 |
| rs1883025 | *ABCA1* | C/T | | 0.13 | | 0.71 |
| rs2000999 | *HPR* | G/A | | 0.31 | | 0.58 |
| rs2072183 | *NPC1L1* | G/C | | 0.16 | | 0.69 |
| rs2081687 | *CYP7A1* | C/T | | 0.01 | | 0.94 |
| rs2131925 | *ANGPTL3* | T/G | | 0.96 | | 0.33 |
| rs2255141 | *GPAM* | G/A | | 0.01 | | 0.94 |
| rs2277862 | *ERGIC3* | C/T | | 1.66 | | 0.20 |
| rs2290159 | *RAF1* | G/C | | 0.11 | | 0.74 |
| rs2479409 | *PCSK9* | A/G | | 0.1 | | 0.75 |
| rs2642442 | *MOSC1* | T/C | | 1.35 | | 0.25 |
| rs2737229 | *TRPS1* | A/C | | 3.73 | | 0.05 |
| rs2954029 | *TRIB1* | A/T | | 2.13 | | 0.14 |
| rs3177928 | *HLA* | G/A | | 0.25 | | 0.62 |
| rs3757354 | *MYLIP* | C/T | | <0.01 | | 0.95 |
| rs4299376 | *ABCG5/8* | T/G | | 0.11 | | 0.75 |
| rs514230 | *IRF2BP2* | T/A | | 0.98 | | 0.32 |
| rs581080 | *TTC39B* | C/G | | 1.28 | | 0.26 |
| rs6029526 | *TOP1* | T/A | | 0.39 | | 0.53 |
| rs629301 | *SORT1* | T/G | | 1.25 | | 0.26 |
| rs6511720 | *LDLR* | G/T | | 0.03 | | 0.87 |
| rs6882076 | *TIMD4* | C/T | | 0.5 | | 0.48 |
| rs7206971 | *OSBPL7* | G/A | | 0.75 | | 0.39 |
| rs7241918 | *LIPG* | T/G | | 0.05 | | 0.82 |
| rs7515577 | *EVIS* | A/C | | 3.22 | | 0.07 |
| rs7570971 | *RAB3GAP1* | C/A | | 0.08 | | 0.78 |
| rs7941030 | *UBASH3B* | T/C | | 3.28 | | 0.07 |
| rs9488822 | *FRK* | A/T | | 3.66 | | 0.06 |
| rs964184 | *APOA1* | C/G | | 0.43 | | 0.51 |
| rs9987289 | *PPP1R3B* | G/A | | 1.97 | | 0.16 |
| **Triglycerides** | | | | | | |
| rs10195252 | *COBLL1* | T/C | | 0.16 | | 0.69 |
| rs10401969 | *CILP2* | T/C | | 0.9 | | 0.34 |
| rs1042034 | *APOB* | T/C | | 2.35 | | 0.13 |
| rs10761731 | *JMJD1C* | A/T | | 0.5 | | 0.48 |
| rs11613352 | *LRP1* | C/T | | 0.32 | | 0.57 |
| rs11649653 | *CTF1* | C/G | | 0.29 | | 0.59 |
| rs1169288 | *HNF1A* | A/C | | 1.75 | | 0.19 |
| rs1260326 | *GCKR* | C/T | | 2.8 | | 0.09 |
| rs12678919 | *LPL* | A/G | | 0.01 | | 0.93 |
| rs13238203 | *TYW1B* | C/T | | 0.25 | | 0.62 |
| rs1532085 | *LIPC* | G/A | | 0.04 | | 0.84 |
| rs17145738 | *MLXIPL* | C/T | | 1.29 | | 0.26 |
| rs174546 | *FADS1-2-3* | C/T | | 0.02 | | 0.90 |
| rs2068888 | *CYP26A1* | G/A | | 3.01 | | 0.08 |
| rs2247056 | *HLA* | C/T | | 1.59 | | 0.21 |
| rs2131925 | *ANGPTL3* | T/G | | 2.42 | | 0.12 |
| rs2412710 | *CAPN3* | G/A | | 0.08 | | 0.78 |
| rs2929282 | *FRMD5* | A/T | | 0.57 | | 0.45 |
| rs2954029 | *TRIB1* | A/T | | 0.25 | | 0.62 |
| rs2972146 | *IRS1* | T/G | | 0.18 | | 0.67 |
| rs3764261 | *CETP* | C/A | | 0.36 | | 0.55 |
| rs439401 | *APOE* | C/T | | 2.44 | | 0.12 |
| rs442177 | *KLHL8* | T/G | | 0.01 | | 0.93 |
| rs4846914 | *GALNT2* | A/G | | 0.04 | | 0.85 |
| rs5756931 | *PLA2G6* | T/C | | 0.04 | | 0.84 |
| rs6065906 | *PLTP* | T/C | | 0.25 | | 0.61 |
| rs645040 | *MSL2L1* | T/G | | 2.85 | | 0.09 |
| rs6882076 | *TIMD4* | C/T | | 0.43 | | 0.51 |
| rs9686661 | *MAP3K1* | C/T | | 0.43 | | 0.51 |
