# Supplementary material for: Variants Identified in a GWAS Meta-Analysis for Blood Lipids Are Associated with the Lipid Response to Fenofibrate
Source: PLoS One. 2012 Oct 31;7(10):e48663. doi: 10.1371/journal.pone.0048663 (PMC3485381; doi:10.1371/journal.pone.0048663)
Supplement: Table S3 — Associations between selected genetic polymorphisms and lipid concentrations at baseline. (DOC) [file pone.0048663.s004.doc]

| **SNP** | **Locus** | | **F** | **Discovery**  **P-value** | **Replication P-value**  **(if genotyped)** | | |
| --- | --- | --- | --- | --- | --- | --- | --- |
| **High-density lipoprotein cholesterol** | | | | | | | |
| rs3764261 | *CETP* | | 26.5 | <0.0001 | 0.0007 | | |
| rs1532085 | *LIPC* | | 13.2 | 0.0003 | -- | | |
| rs964184 | *APOA1* | | 9.2 | 0.003 | 0.00005 | | |
| rs7134594 | *MVK* | | 7.5 | 0.006 | -- | | |
| rs737337 | *LOC55908* | | 6.8 | 0.009 | -- | | |
| rs17145738 | *MLXIPL* | | 6.6 | 0.01 | -- | | |
| rs1883025 | *ABCA1* | | 6.1 | 0.01 | -- | | |
| rs605066 | *CITED2* | | 5.9 | 0.02 | -- | | |
| rs9987289 | *PPP1R3B* | | 5.2 | 0.02 | -- | | |
| rs16942887 | *LCAT* | | 4.3 | 0.04 | -- | | |
| **Low-density lipoprotein cholesterol** | | | | | |  | |
| rs629301 | *SORT1* | 13.6 | | 0.0002 | -- | | |
| rs6511720 | *LDLR* | 9.0 | | 0.003 | -- | | |
| rs9987289 | *PPP1R3B* | 8.6 | | 0.003 | -- | | |
| rs1367117 | *APOB* | 8.3 | | 0.004 | -- | | |
| rs174546 | *FADS1-2-3* | 6.8 | | 0.01 | -- | | |
| rs2081687 | *CYP7A1* | 5.7 | | 0.02 | -- | | |
| rs12670798 | *DNAH11* | 5.6 | | 0.02 | -- | | |
| rs7941030 | *UBASH3B* | 5.0 | | 0.03 | -- | | |
| rs3764261 | *CETP* | 4.4 | | 0.04 | 0.27 | | |
| **Total cholesterol** | | | | | |  | |
| rs629301 | *SORT1* | 14.2 | | 0.0002 | -- | | |
| rs9987289 | *PPP1R3B* | 10.7 | | 0.001 | -- | | |
| rs3764261 | *CETP* | 10.2 | | 0.001 | 0.09 | | |
| rs174546 | *FADS1-2-3* | 9.8 | | 0.002 | -- | | |
| rs10128711 | *SPTY2D1* | 7.8 | | 0.005 | -- | | |
| rs1367117 | *APOB* | 6.1 | | 0.01 | -- | | |
| rs7941030 | *UBASH3B* | 4.6 | | 0.03 | -- | | |
| rs1532085 | *LIPC* | 4.5 | | 0.03 | -- | | |
| rs12670798 | *DNAH11* | 4.4 | | 0.04 | -- | | |
| **Triglycerides** | | | | | | |  |
| rs964184 | *APOA1* | 31.2 | | <0.0001 | 0.0003 | | |
| rs2131925 | *ANGPTL3* | 9.6 | | 0.002 | -- | | |
| rs17145738 | *MLXIPL* | 7.8 | | 0.005 | -- | | |
| rs2972146 | *IRS1* | 4.5 | | 0.04 | -- | | |
