# Supplementary material for: Variants Identified in a GWAS Meta-Analysis for Blood Lipids Are Associated with the Lipid Response to Fenofibrate
Source: PLoS One. 2012 Oct 31;7(10):e48663. doi: 10.1371/journal.pone.0048663 (PMC3485381; doi:10.1371/journal.pone.0048663)
Supplement: Text S1 — Detailed description of genotyping and circulating blood lipid measurement in the GOLDN and HyperTG studies. (DOC) [file pone.0048663.s005.doc]

*Genotyping*
DNA extraction and purification in the GOLDN study has been described in detail in a previous manuscript from our group [15]. We genotyped 906,600 SNPs using the Affymetrix Genome-Wide Human 6.0 array and the Birdseed calling algorithm [21]. Of the 86 loci identified to be associated with baseline lipids in Caucasians by Teslovich et al. [1], 15 were available on the array and the rest were imputed using MACH software (Version 1.0.16, University of Michigan, Ann Arbor, MI) with the Human Genome Build 36 as the reference. Missing typed data were kept as missing in the final genotype data set. Population substructure was assessed using principal components generated using EIGENSOFT 3.0 and found to be limited in the GOLDN data [23]. The first 10 principal components were retained and tested for association with the lipid outcomes; no statistically significant associations were found. As our statistical models also adjusted for pedigree, confounding due to population stratification was not a major threat to validity of our findings.

Among the 7 SNPs identified by GOLDN, rs2642442, rs964184, rs4420638 were genotyped using the Taqman assay (Applied Biosystems) for 267 HyperTG samples, and rs10401969, rs3764261, rs492602 and rs7134375 genotype data were obtained from the Metabochip (Voight BF, Kang HM, Ding J, Palmer CD, Sidore C, *et al*. (2012) The metabochip, a custom genotyping array for genetic studies of metabolic, cardiovascular, and anthropometric traits. PLoS Genet 8: e1002793). The family pedigree structure was examined by Prest-plus (v4.09). Potential population substructure was assessed using principal components generated by EIGENSOFT 3.0. The first 20 principal components were tested and no statistically significant associations were found. The statistical model was adjusted for pedigree.

*Lipid Measurements*

Circulating blood lipids were ascertained at baseline and following the three-week fenofibrate treatment in GOLDN participants. Samples were collected and processed using a standardized protocol, aliquoted, and stored at -70 degrees C until the end of the study. All samples for each individual were analyzed in the same batch to minimize variability. LDL cholesterol was measured using a homogeneous direct method (LDL Direct Liquid Select™ Cholesterol Reagent, Equal Diagnostics, Exton, PA) on a Hitachi 911 Automatic Analyzer [24]. Triglycerides were measured using the glycerol-blanked enzymatic method on the Roche COBAS FARA analyzer (Roche Diagnostics Corporation, Basel, Switzerland) [24]. HDL cholesterol was measured using the same method as triglycerides following precipitation of non-HDL cholesterol with magnesium/dextran [24].

In HyperTG, circulating blood lipids were ascertained at baseline and at the follow-up visit after the participants were treated with fenofibrate 160 mg per day for eight weeks. Plasma triglyceride, total cholesterol and direct HDL cholesterol concentrations were determined by enzymatic colorimetric assays using a Chemistry Analyzer Model ATAC 8000 (Elan Diagnostics, Brea, CA). For subjects with triglyeride levels below 400 mg/dL, LDL cholesterol was calculated using the Friedewald equation (LDL cholesterol = total cholesterol – HDL cholesterol - 0.2*triglycerides).
